# Polymethylmethacrylate-assisted ventral discectomy: Rate of pseudarthrosis and clinical outcome with a minimum follow-up of 5 years

**DOI:** 10.1186/1471-2474-12-140

**Published:** 2011-06-28

**Authors:** Mario Cabraja, Daniel Koeppen, Wolfgang R Lanksch, Klaus Maier-Hauff, Stefan Kroppenstedt

**Affiliations:** 1 Department of Neurosurgery, Charité-Universitätsmedizin Berlin, Berlin, Germany

## Abstract

**Background:**

Polymethylmethacrylate (PMMA) assisted ventral discectomy has been criticized for high rates of graft migration and pseudarthrosis when compared with various other fusion procedures for the treatment of cervical degenerative disc disease (DDD), therefore rendering it not the preferred choice of treatment today. Recently however spine surgery has been developing towards preservation rather than restriction of motion, indicating that fusion might not be necessary for clinical success. This study presents a long term comparison of clinical and radiological data from patients with pseudarthrosis and solid arthrodesis after PMMA assisted ventral discectomy was performed.

**Methods:**

From 1986 to 2004 416 patients underwent ventral discectomy and PMMA interposition for DDD. The clinical and radiological outcome was assessed for 50 of 127 eligible patients after a mean of 8.1 years. Based on postoperative radiographs the patients were dichotomized in those with a pseudarthrosis (group A) and those with solid arthrodesis (group B).

**Results:**

Pseudarthrosis with movement of more than 2 of the operated segment was noted in 17 cases (group A). In 33 cases no movement of the vertebral segment could be detected (group B). The analysis of the clinical data assessed through the neck disability index (NDI), the visual analogue scale (VAS) of neck and arm pain and Odom's criteria did not show any significant differences between the groups.

Patients from group B showed a trend to higher adjacent segment degeneration (ASD) than group A (p = 0.06). This correlated with the age of the patients.

**Conclusions:**

PMMA assisted discectomy shows a high rate of pseudarthrosis. But the clinical long-term success does not seem to be negatively affected by this.

## Background

Ventral microdiscectomy and fusion are currently the golden standard in surgical treatment of degenerative disc disease (DDD)[1-3]. There is nevertheless an ongoing discussion as to which fusion substrate provides the best clinical and radiological outcome. Autologous iliac bone crest, allograft bone, titanium, polyetheretherketone (PEEK) or carbon cages are widely used graft materials. Polymethylmethacrylate (PMMA) is another substitute for removed discs [4], but there have been reports of higher rates of graft migration and pseudarthrosis for PMMA compared to carbon- or titanium cages [5-7]. Another major concern with using PMMA in ventral discectomy is unsatisfying results in restoring disc height and sagittal alignment [1,7,8]. Nevertheless, PMMA is an economic alternative to titanium and PEEK implants [9,10].

The concept of spinal fusion in the treatment of DDD is currently being questioned as recent developments in spinal surgery move towards preservation rather than restriction of motion. Therefore, PMMA's poor results in regard to pseudarthrosis might be of much less relevance for the clinical outcome as previously assumed.

The aim of the present study was to evaluate the clinical outcome of patients suffering from DDD with pseudarthrosis (group A) and solid arthrodesis (group B) following PMMA-assisted discectomy and compare the degeneration of the adjacent segments and sagittal alignment of both groups in a long term follow-up.

## Methods

**Figure 1:**
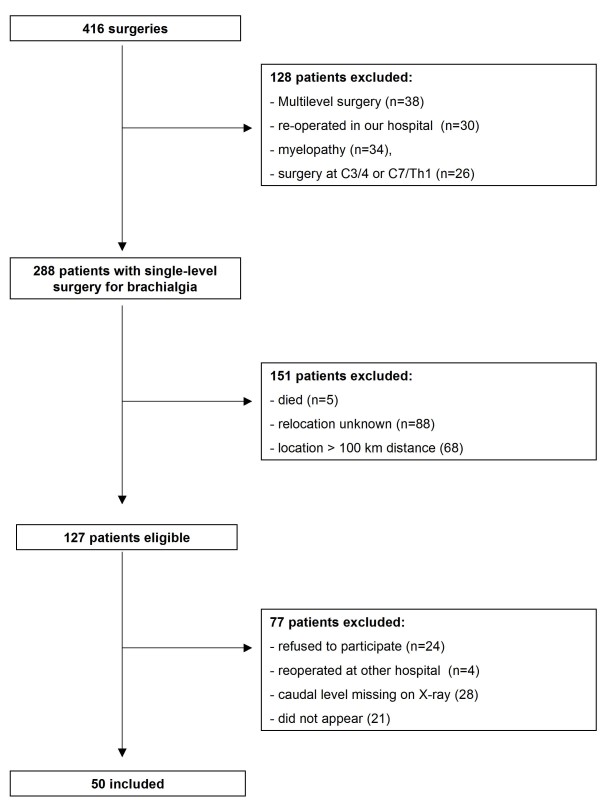
**Patient flow**.

From 1986 to 2004 a total of 416 patients underwent ventral discectomy and PMMA interposition. The surgical procedures were performed by two senior neurosurgeons. For better clinical comparison patients suffering from myelopathy or traumatic spinal cord injury were excluded for the purpose of this study. To evaluate adjacent segments, patients operated on segments C3-4 and C7-T1 were excluded from the study (see Figure 1) as well.

The research was conducted conforming to the Helsinki Declaration as well as local legislation. By ensuring the patient's anonymity approval from our institution's ethics committee is not required for a retrospective study.

The included patients described radiculopathy and neck pain as their main symptoms. The patients did not profit from conservative treatment. Radiographic examinations included plain radiography, MRI, CT and myelography.

The ventral discectomy was performed in supine position by a transverse skin incision from the right side after induction of general anaesthesia. After disc removal and decompression the interspace was drilled with a 5 mm drill under anterior distraction to create a groove in the middle part of each endplate. This was done to embed the PMMA in the disc space to prevent graft dislocation. Before application of PMMA a gelatine foam was placed in the disc space to protect the dura and nerve roots. Then the PMMA (Palacos^® ^R, Biomet Merck, and Refobacin^® ^Bone Cement R, Biomet Europe Group, Dordrecht, The Netherlands; Sulzer Comp., Baar, Switzerland) was inserted into the disc space with a syringe. The situs was rinsed with saline solution until the PMMA hardened. Afterwards the interbody distraction was released.

Post surgery the patients were all treated with the same protocol which consisted of physical rest for 6 weeks and then physical therapy.

Clinical examination and radiographs including plain and flexion-extension films were performed on an outpatient basis in our department. Neck and arm pain were measured by Visual Analogue Scale (VAS); functionality was assessed by the Neck Disability Index (NDI). General clinical outcome was rated using Odom's criteria.

Radiological analysis involved the measurement of three angles. The cervical lordosis was measured between C2 and C7 according to Cobb in neutral position as well as in extension and flexion. The segmental angles of the operated and adjacent vertebral levels were also measured in neutral position and in extension and flexion.

Adjacent segment degeneration (ASD) was defined as changes on plain radiographs at segments adjacent to the previously operated [11] and disc height reduction compared to a neighboring unimpaired disc [12,13].

We dichotomized the patients in two groups so as to best compare clinical and radiological outcome: Movement of more than 2 shown on flexion/extension radiographs and/or the presence of radiolucency around the graft was regarded as pseudarthrosis (group A) [2,3]. The absence of motion between the spinous process and the bodies shown on flexion-extension lateral radiographs of the operated segment was rated as solid arthrodesis (group B) according to accepted criteria [3,14].

The statistical evaluation was performed using PASW Statistics 18, Version 18.0.0 (SPSS Inc.). Statistical analysis of ASD and gender was performed by Pearson's chi-square test. The clinical and radiological data were analysed by the Mann-Whitney-U-test and the Student's t-test. A p-value < 0.05 was deemed as statistically significant.

## Results

50 of 127 eligible patients (22 men and 28 women) were evaluated. The patients' age at time of operation ranged from 44 to 79 years with a mean of 58.1 years.

Ventral discectomies were performed in 5 cases on C4/5, in 29 cases on C5/6 and in 16 cases on C6/7. The follow-up period ranged from 61 to 250 months (mean: 98 months). Implantation of the PMMA grafts was performed without complications. Intra- or postoperative graft dislocation did not occur.

Pseudarthrosis was observed in 17 cases (34%, group A). In 33 cases no movement could be found (66%, group B).

Both groups showed no differences in sagittal cervical and segmental lordosis (p > 0.08). In accordance with our criteria the patients of group B did not demonstrate any movement of the operated segment, while in group A a movement of 5.8 ± 0.7 was measured. ROM of the cervical spine was significantly higher in group A (p = 0.008) (Table 1). ROM of the adjacent segments did not differ significantly between the two groups (p = 0.1).

**Table 1:** Cervical Lordosis and Range of Motion

	Group A	Group B	p-value
**C2-7, plain**	10.3 ± 2.9	9.2 ± 2.2	0.774
**Operated segment, plain**	2.7 ± 0.6	1.2 ± 0.5	0.082
**C2-7 ROM**	43.0 ± 3.1	33.7 ± 1.8	0.008
**Operated segment ROM**	5.8 ± 0.7	0 ± 0	< 0.0001
**cranial segment ROM**	10.3 ± 1.0	8.0 ± 0.9	0.110
**caudal segment ROM**	9.5 ± 1.2	7.1 ± 0.7	0.084

Mean lordosis angle in plain scans [°] and mean sagittal range of motion (ROM) in flexion-extension scans [°] with SEM.

Patients of group B (59.8 ± 9.5 years) were significantly older than the patients of group A (54.4 ± 8.2 years) (p = 0.027). The interval between surgery and assessment for the sake of this study was significantly longer (p = 0.034) in group B (109.6 ± 43.5 months) than in group A (80.8 ± 34.9 months) (Table 2).

**Table 2:** Adjacent Segment Degeneration

	ASD	no ASD	Age (years)	Time interval (months)
**Group A (n = 17)**	9 (52.9%)	8 (47.1%)	54.4 ± 8.2	80.8 ± 34.9
**Group B (n = 33)**	26 (78.8%)	7 (21.2%)	59.8 ± 9.5	109.6 ± 43.5
**p-value**	0.06	0.06	0.027	0.034

Patients with and without adjacent segment degeneration (ASD) dichotomized in patients with pseudarthrosis (group A) and solid arthrodesis (group B) of the operated segment.

**Figure 2:**
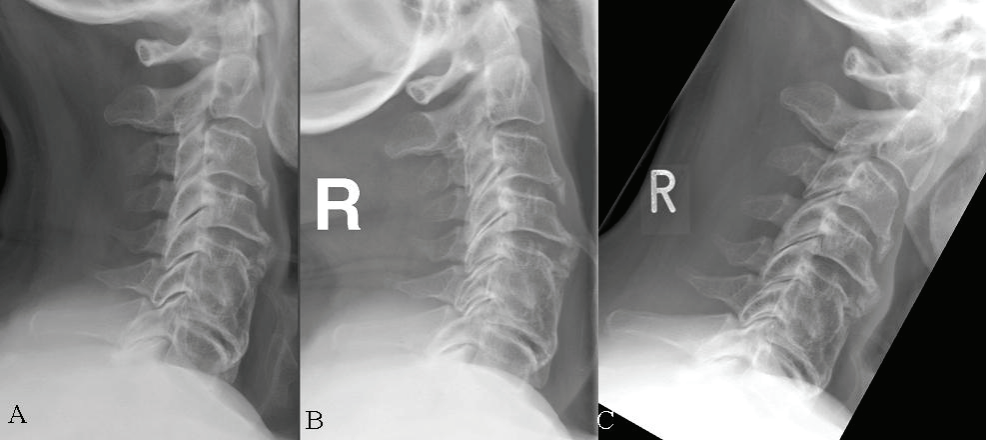
**Exemplary plain (A), extension (B) and flexion (C) radiographs of a 67 years old patient with achieved fusion and ASD 7 years after surgery at level C5/6**. In figure 1 a multisegmental degeneration can be seen.

**Figure 3:**
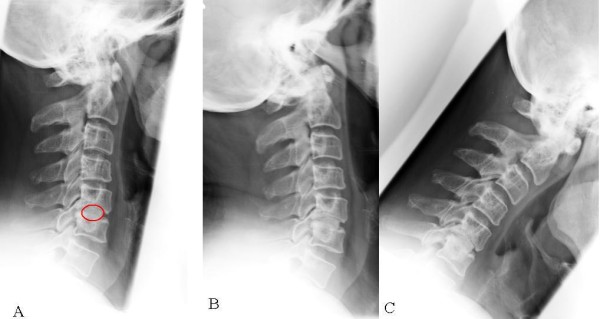
**Exemplary plain (A), extension (B) and flexion (C) radiographs of a 57 year old patient with pseudarthrosis of the operated level and without signs of ASD 7 years after surgery**. The operated pseudarthrotic level C5/6 is indexed, and the spheric form of the graft material has the aim to prevent migration (A).

ASD could be found in 35 cases (70%) (Figure 2) with 9 patients belonging to group A (25.7%) and 26 patients to group B (74.3%) (Figure 3). In 15 cases (30%) no signs of ASD was found using radiological imaging, 8 of these 15 cases belonging to group A (46.7%), 7 to group B (53.3%). Statistical analysis showed a trend (p = 0.06) when comparing signs of ASD in both groups (Table 2). The patients' age had a significantly higher impact on the development of ASD (p = 0.011) than the motion of the operated segment (p = 0.06).

60.6% (n = 20) of group B showed an excellent or good outcome. In group A 76.5% (n = 13) of the patients rated an excellent or good clinical outcome according to Odom's criteria, although the analysis of the clinical data did not show any significant differences in the two groups when assessed by NDI (p = 0.418), VAS (p > 0.346) and Odom's criteria (p = 0.18) (Tables 3 and 4). Comparing the clinical outcome (NDI, VAS and Odom's) of patients with signs of ASD and those without signs of ASD did not reveal any significant clinical differences either (p > 0.527).

**Table 3:** Clinical Outcome

	Group A	Group B	p-value
**NDI**	11.6 ± 1.6	13.7 ± 1.6	0.418
**VAS neck**	3.5 ± 0.6	2.8 ± 0.4	0.346
**VAS right arm**	1.3 ± 0.5	1.8 ± 0.4	0.40
**VAS left arm**	2.1 ± 0.6	1.8 ± 0.4	0.678

Clinical outcome of both groups assessed by the neck disability index (NDI) and visual analogue scale (VAS).

**Table 4:** Odom's Criteria

	Group A	Group B
**Excellent**	8 (47.1%)	12 (36.4%)
**Good**	5 (29.4%)	8 (24.2%)
**Fair**	4 (23.5%)	8 (24.2%)
**Poor**	0	5 (15.2%)

Overall clinical outcome according to Odom's criteria of both groups.

## Discussion

We present a retrospective long-term follow-up study of patients with a high rate (34%) of pseudarthrosis after PMMA-assisted ventral discectomy. No statistically relevant differences in clinical outcome could be found in patients with pseudarthrosis (group A) versus patients with solid arthrodesis (group B) of the operated segment. Signs of ASD could be detected more often in the group of patients with solid arthrodesis (group B), but the appearance of ASD correlated rather with the patients' age than with the movement of the operated segment.

### Pseudarthrosis and PMMA

The high rate of failed fusion and pseudarthrosis (ranging from 54-98%) is regarded as the major disadvantage of PMMA [7,8,15,16]. The bone cement in the disc space does not allow a complete bony fusion. Taking this into account our results still go conform with prior studies that the clinical outcome of patients undergoing ventral discectomy and PMMA-assisted fusion does not seem to differ substantially from patients treated with other substrates [1,5,7,8,17]. An adequate fusion can also be achieved with filled PMMA-cages [18].

The existing literature (see table 5) dealing with PMMA-assisted cervical discectomy lacks data on segmental and cervical motion and the situation of the adjacent segments. ROM of the operated segment in group A of our study (5.8) was almost comparable to physiologic ROM [19-22]. Pseudarthrosis of the operated segments does not seem to influence the adjacent segments negatively.

**Table 5:** Overview of PMMA-studies in the last 20 years

Author, Year	Study design, follow-up	Material	No. of patients	Clinical outcome	Fusion according to author	Re-OP
**Böker et al., 1989 **[15]	Retrospective 15-20 years	PMMA	57	not assessed	89%	0
**Samii et al., 1989**[16]	Retrospective 4.8 years	PMMA	438	81% pain relief	not assessed	35 (8%)
**van den Bent et al., 1996**[7]	Prospective 2 years	PMMA MDO	42 39	70% good 77% good	28% 63%	0 0
**Hamburger et al., 2001**[8]	Retrospective 12.2 years	PMMA	249	77.5% exc. or good	53.8%	24 (9.6%)
**Jöllenbeck et al., 2001**[10]	Prospective 7 days	PMMA TTC	100 100	81% compl. recovery 78% "	not assessed	1 (1%) 1 (1%)
**Bärlocher et al., 2002**[1]	Prospective 1 year	MDO ABG PMMA TTC	33 30 26 36	75.5% exc. or good 80% " 87.5% " 94.4% "	93.3% 65.3% 0% 97.2%	2 (6.1%) 1 (3.3%) 0 0
**Chen et al., 2005**[18]	Prospective 2 years	PMMA-cage	63	100% exc. or good	100%	0
**Korinth et al., 2006**[17]	Retrospective 6 years	PMMA	124	93.6% exc. or good	not assessed	3 (2.4%)
**Schröder et al., 2007**[5]	Prospective 2 years	PMMA TTC	53 54	85% exc. or good 77.7% "	66% 87%	0 0

Overview of studies in the last 20 years evaluating the outcome of patients with DDD treated with ventral discectomy and PMMA-assisted fusion.

MDO = Microdiscetomy only; ABG = autologous bone graft; TTC = titanium cage

### Cervical alignment

Good or excellent clinical outcome can be achieved for the majority of cases by anterior cervical discectomy alone without fusion [23]. But this procedure is associated with longer postoperative neck pain [24]. Furthermore a kyphotic cervical alignment might develop or, in case of preoperative kyphotic curvature, lordotic alignment cannot be achieved [25]. These points will be critically analyzed in a prospective randomized multicenter trial (NECK-trial)[26]. The use of PMMA for fusion has been criticized to have a similar disadvantage as the intervertebral space can only be filled by PMMA and does not allow an intervertebral fusion. In our study sample a lordotic angle of the operated segment could be found in the vast majority of cases. It is to be noted that the disc space was distracted anterior before PMMA filling and released only after end of the PMMA hardening process. Thus lordotic alignment can be achieved with PMMA by anterior distraction and placement of the PMMA in the anterior or in the middle part.

The presented study however lacks preoperative data. It is not known if the lordotic posture of the cervical spine increased, decreased or was maintained from its status before the operation. However, the postoperative cervical lordosis of approximately 10 in both our study groups does not differ from patients that undergo fusion surgery or cervical arthroplasty [20,27].

Graft dislocation and graft breakage [5-7,25,28] have not been found during the course of our long-term study. The described technique of embedding the PMMA offers a possible explanation for these results.

### Clinical outcome

Whether osseous fusion is necessary for a favourable outcome after cervical discectomy is subject of a controversial discussion [25]. Out study shows that PMMA does not offer inferior long term clinical results even though segmental fusion is inferior compared to other allograft substitutes [1].

Our comparably low rate of excellent and good outcome (60.6%) can be explained with a significantly longer follow-up period compared to most of the listed studies (Table 5). We also differentiated between arm and neck pain. Our patients experienced substantial relief of their brachialgia, but still complained of neck pain (see table 3), which could be related to the natural course of the degenerative disease.

The findings of ASD did not affect clinical outcome.

### Range of motion

Despite a longer follow-up period the cervical C2-7 ROM of our study groups (Group A 43.0, Group B 33.7) was almost comparable to patients undergoing fusion surgery or arthroplasty in prospective studies 2 years after surgery (39.6 after fusion and 53.2 after arthroplasty)[29,30].

Due to different kinematics of the upper cervical spine (C1-3) and our specifically determined interest in ASD of cranial as well as caudal levels patients operated on C3-4 and C7/T1 were excluded from our study.

25% to 92% of patients undergoing cervical surgery develop ASD within 4.5 to 8 years [12,31-33]. The high prevalence of ASD 8 years after surgery in our group B goes conform with these findings of previous studies, but might be a consequence of a higher preoperative prevalence as well. It is also to be noted that the patients of our group B were significantly older (5.4 years) and were examined after a significantly longer period (28.8 months) following surgery. In contrast to previous studies the detection of ASD in our study did not correlate with the time interval between surgery and last follow-up [12], but only with the age of the patients.

The prevalence of adjacent segment disease ranges from 9 to 14% following fusion [8,32,34-37]. Our overall collective (n = 416) comprised of 34 patients (8.2%) that had been excluded because of the need for surgery of an adjacent level. It is not known if the previously operated segment of these patients was pseudarthrotic or solid before the second surgery.

Preoperative plain or functional imaging was available only in a small number of our patients. Therefore our study lacks preoperative imaging of the cervical spine for better comparison with the postoperative radiological status.

Whether ASD or adjacent segment disease is a consequence of previous stabilizing surgery or the natural course of the disease [33,38] as indicated by the higher age and longer follow-up period of our group B remains controversial.

## Conclusions

Ventral discectomy and fusion with PMMA represents a cheap, safe and successful procedure in the treatment of cervical DDD. Although the rate of pseudarthrosis following PMMA-assisted cervical surgery is comparably high, clinical outcome does not differ from that of patients with successful solid arthrodesis. Furthermore we could show that cervical lordosis can be achieved with PMMA by distraction of intervertebral space. This data might justify a randomized multicenter study with PMMA as a substrate alongside recently more accepted materials as PEEK, carbon and titanium.

## Abbreviations

ABG: autologous bone graft; ASD: adjacent segment degeneration; DDD: degenerative disc disease; MDO: Microdiscetomy only; NDI: neck disability index; PEEK: polyetheretherketone; PMMA: polymethylmethacrylate; ROM: range of motion; SEM: standard error of the mean; TTC: titanium cage; VAS: visual analogue scale.

## Competing interests

The authors declare that they have no competing interests.

## Authors' contributions

MC was responsible for conception, design, data analysis, writing and editing of the MS. DK was responsible for data analysis, writing and editing of the MS, WL was responsible for data analysis, writing and editing, KM was responsible for data analysis, writing and editing, SK was responsible for conception, design, data analysis, writing and editing of the MS. All authors read and approved the final manuscript.

## Pre-publication history

The pre-publication history for this paper can be accessed here:

http://www.biomedcentral.com/1471-2474/12/140/prepub
